# CaMKIV mediates spine growth deficiency of hippocampal neurons by regulation of EGR3/BDNF signal axis in congenital hypothyroidism

**DOI:** 10.1038/s41420-022-01270-4

**Published:** 2022-12-06

**Authors:** Hui Wu, Guihai Suo, Tianci Li, Yuqin Zheng, Haiying Li, Feifei Shen, Yongjun Wang, Haidong Ni, Youjia Wu

**Affiliations:** 1 Department of Pediatrics, Affiliated Hospital of Nantong University, 20 Xi Si Road, 226001 Nantong, Jiangsu Province China; 2 Department of Pediatrics, The Sixth People’s Hospital of Nantong, Affiliated Nantong Hospital of Shanghai University, 226001 Nantong, Jiangsu Province China; 3 Key Laboratory of Neuroregeneration of Jiangsu and Ministry of Education, Co-innovation Center of Neuroregeneration, Nantong University, 226001 Nantong, Jiangsu Province China

**Keywords:** Developmental disorders, Axon and dendritic guidance

## Abstract

Congenital hypothyroidism (CH) will cause cognitive impairment in the condition of delayed treatment. The hippocampus is one of the most affected tissues by CH, in which the functional structures of hippocampal neurons manifest deficiency due to aberrant expression of effector molecules. The Ca^2+^/Calmodulin-dependent protein kinase, CaMKIV, is downregulated in the hippocampal neurons, influencing the growth of dendritic spines in response to CH. However, the underlying mechanism is not fully elucidated. In the present study, the early growth response factor 3 (EGR3) was regulated by CaMKIV in the hippocampal neurons of CH rat pups, as was analyzed by transcriptome sequencing and in vitro cell experiments. EGR3 localized within hippocampal neurons in CA1, CA3, and dentate gyrus regions. Deficient EGR3 in the primary hippocampal neurons significantly reduced the density of dendritic spines by downregulating the expression of BDNF, and such effects could be rescued by supplementing recombinant BDNF protein. Taken together, CH mediates cognitive impairment of pups through the inactivation of CaMKIV in the hippocampal neurons, which decreases the expression of EGR3 and further reduces the production of BDNF, thereby impairing the growth of dendritic spines. Identifying CaMKIV/EGR3/BDNF pathway in the hippocampal neurons in the context of CH will benefit the drug development of intellectual disability caused by CH.

## Introduction

Congenital hypothyroidism (CH) is one of the common hereditary diseases in pediatrics. Although thyroid hormone (TH) can be transferred to the fetus through the placenta, children still suffer from CH in a certain proportion without a comprehensive screening of neonatal diseases. CH always results in a deficiency of physical growth, intellectual development, and other physiological function [1]. The typical pathological feature of CH is attributed to insufficient TH, which leads to abnormal development and metabolism of tissues and organs [2]. It is widely known that thyroid dysplasia accounts for 85% of primary CH in iodine-sufficient countries, while 10–15% are thyroxine synthesis or thyroxine transport metabolism disorder [3].

TH, especially T3, plays an important role in promoting the migration and differentiation of nerve cells, synaptic formation, and myelination [4]. T3 can activate Ca^2+^/calmodulin-dependent protein kinase type IV (CaMKIV) of cortical and hippocampal neurons in a concentration- and time-dependent manner [5–7], which leads to rapid phosphorylation of cAMP-response element binding protein (CREB), thereafter influences neurogenesis, neuronal survival, and synaptic plasticity [8]. Our previous works have shown that CH-mediated downregulation of CaMKIV in dentate gyrus (DG) neurons results in deficient growth of dendritic spines through regulation of CREB, which impairs the cognitive behaviors of the offspring [9]. However, the regulatory mechanisms of CaMKIV on CH-mediated cognitive deficits are not fully elucidated.

Immediate early genes (IEGs) are the first activated target genes in a variety of cells in response to extracellular and intracellular signal stimulation [10–13]. Especially, the Ca^2+^ influx signal, which is also associated with the activation of CaMKs, is closely related to the enhanced transcriptional activity of IEGs in the neurons [14]. Early growth response factor (EGR), a member of IEGs, contains four family members, including EGR1, EGR2, EGR3, and EGR4 [15]. EGR3 is abundantly detectable in the neurons of the cerebral cortex, hippocampus, DG, caudate nucleus, amygdala, and other brain regions [16, 17]. Aberrant expression of EGR3 has been shown to impair synaptic activity [18, 19], dendrite morphogenesis [20], and volume and fiber bundle connections in the hippocampus [21], thus associated with the pathogenesis of several neuropsychiatric diseases, including schizophrenia [21] and seizures [22, 23]. The EGR3-mediated pathological effects on hippocampal neurons are implicated in the regulation of brain-derived neurotrophic factor (BDNF) [11, 24], which plays important roles in the structure and function of excitatory or inhibitory synapses [25]. However, there are still some debates regarding the positive-feedback function of BDNF on the expression of EGR3 [11]. To date, whether the CH-mediated functional deficit of hippocampal neurons is associated with the dysregulation of EGR3 is still unknown.

Given that CH alters Ca^2+^ influx into neurons of the cortex and hippocampus of developing rats [26, 27], which is involved in the inactivation of CaMKIV [28, 29], it is assumed that the EGR3/BDNF axis may be affected by the dysregulation of CaMKIV, as the calmodulin-dependent protein kinase is proved to be active in the regulation of EGRs [30]. Therefore, the CaMKIV/EGR3 signal axis is possibly associated with CH-mediated deficient hippocampus development. The present study examined the dendritic spine growth of hippocampal neurons in CH rat offspring. The expression of EGR3 in the dentate gyrus of the CH rat hippocampus and its correlation with CaMKIV was further analyzed. Decreased expression of EGR3 significantly reduced the density of dendritic spines, which could be rescued by BDNF supplement. Our results demonstrate that the CaMKIV/EGR3 signal axis is responsible for regulating CH-mediated cognitive impairment, which provides a novel clue for the drug development of CH.

## Results

### CH impairs brain development of rat with a reduction of dendritic spines density of hippocampal dentate gyrus neurons

**Fig. 1:**
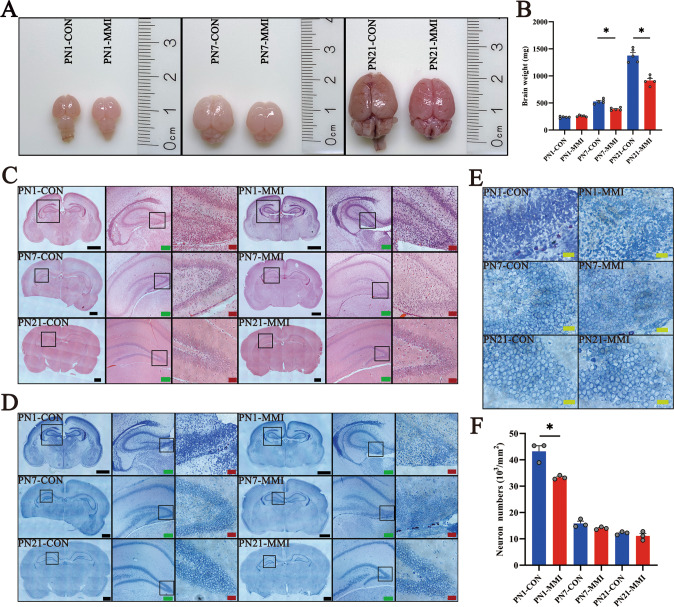
Comparison of cerebral histomorphology between rats with or without CH. **A**, **B** The volume and weight of brain tissue of rat pups with or without CH, *n* = 5. **C** HE staining of the morphology of the hippocampus in the control and CH group. **D**–**F** Nissl’s staining of the hippocampal morphology and the number of neurons in the dentate gyrus of rat pups. The rectangle indicates the magnified region. Statistical analysis of neuron number in triplicates every 15 fields. Scale bars: black, 1000 μm; green, 200 μm; red, 50 μm; yellow, 20 μm. Error bars represent the standard error (*P < 0.05).

**Fig. 2:**
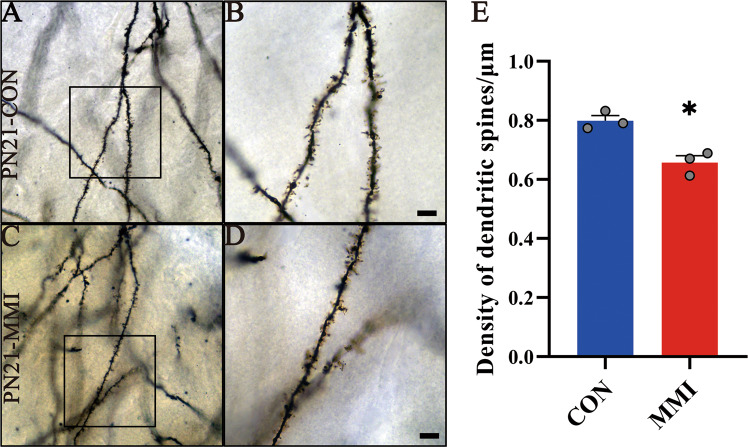
CH results in deficient development of the dendritic spine of hippocampal neurons in PN21 rats. **A**–**D** Comparison of the density of dendritic spines of DGCs between control and CH group of PN21 rats by Golgi staining. The rectangles in (**A**, **C**) indicate the region magnified in (**B**, **D**). **E** statistical analysis of the density of dendritic spines in the control and CH group. Statistical analysis of spine density in triplicates every 15 fields. Scale bar: 25 μm. Error bars represent the standard error (*P < 0.05).

The CH model of rat pups was established by adding 2-mercapto-1-methylimidazole (MMI) into the drinking water of the pregnant dams. We verified the success of the model establishment by detecting the serum TH/TSH levels and the tests of the cognitive function of the offspring [12]. To understand the mechanism of CH-mediated cognitive deficiency, we examined the volume of the brain dissected from CH pups and found that CH significantly affects brain development at postnatal day 7 (PN7) and PN21 (Fig. 1A, B). As CH is known to severely impair the development of hippocampal structure and function [31–33], the hippocampus morphology was then observed following HE staining, which showed undetectable alteration between the CH and the control (Fig. 1C). Subsequently, the number of dentate gyrus neurons in CH rat pups was carefully measured after Nissl’s staining. Statistical analysis demonstrated that CH also had no effects on the number of DG neurons from PN7 onwards, although CH reduced the cell number at PN1 (Fig. 1D–F). Golgi staining was further performed to detect the dendritic spines of DG neurons to investigate CH-mediated impairment of hippocampal development that impacted cognitive function. The dendritic spine density of DG neurons in the CH group was significantly reduced to 82.3% of the control (Fig. 2A–E). The data indicate that CH can impair the pups’ brain development by reducoffing the dendritic spines density of hippocampal dentate gyrus neurons.

### Bioinformatics analysis of hippocampus from CH rat pups during the development

**Fig. 3:**
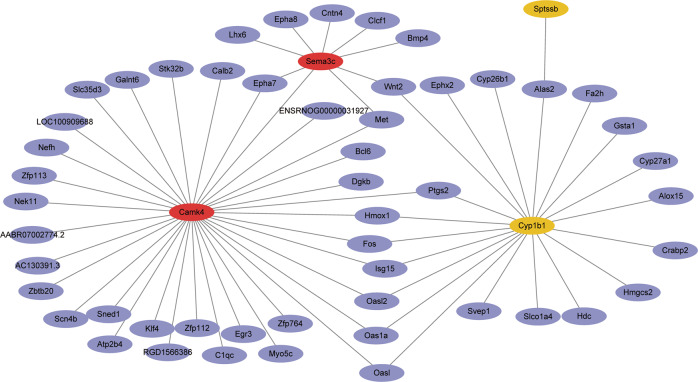
Gene network mediating dendritic spines deficiency in hippocampus of CH rat pups. A gene network was constructed using the Ingenuity Pathway Analysis Software (IPA) based on DEGs at PN1, PN7, and PN21.

To elucidate the regulatory mechanism of CH-mediated aberrant expression of CaMKIV on the dendritic spine growth of hippocampal dentate gyrus neurons, we performed transcriptome sequencing of hippocampus tissues collected from CH and control groups at PN1, PN7, and PN21. 388, 147, and 385 differentially expressed genes (DEGs) were identified, respectively, with the defined standard of *P* < 0.05 and a more or less than twofold change (Supplementary Table S1). These DEGs showed dynamic changes in the hippocampus, as was shown by the heatmap and cluster dendrogram (Supplementary Fig. S1). A gene network was constructed to show the regulatory relationships between CaMKIV and its downstream target gene(s) in the hippocampus of CH rat pups based on the DEGs integrated from three time points. EGR3, together with other downstream target genes, was under the regulation of CaMKIV in the hippocampus of CH rat pups (Fig. 3). The data indicate that CaMKIV/EGR3 may play an important role in the CH-mediated abnormal growth of dendritic spines in hippocampal development of rat pups.

### CaMKIV/EGR3 were downregulated in the hippocampus of CH rat pups

**Fig. 4:**
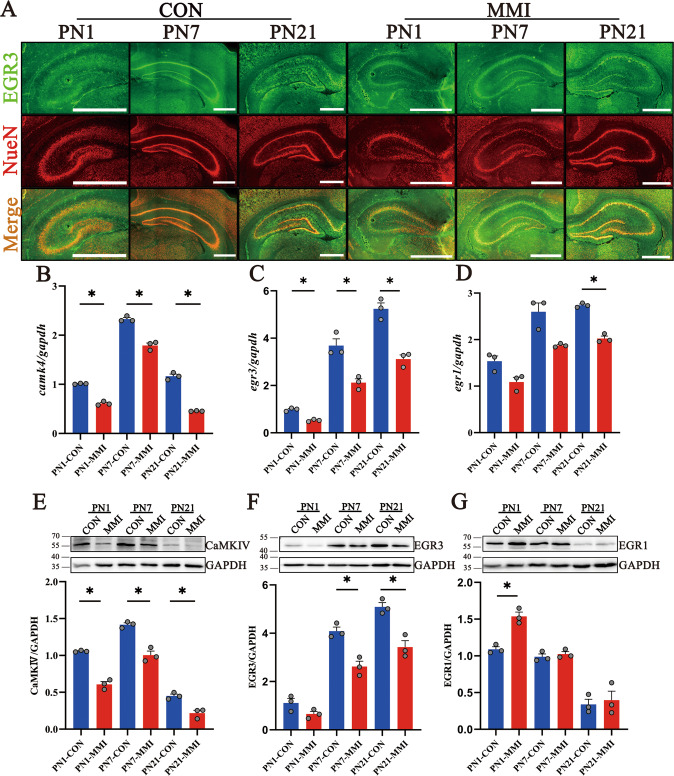
CaMKIV/EGR3 was downregulated in the hippocampus of CH offspring. **A** The co-localization of EGR3 and NeuN-positive neurons in the hippocampus by immunofluorescence staining. CON indicates control, and MMI indicates CH. Scale bar: 1000 μm. **B**–**D** The transcriptional levels of CaMKIV, EGR3, and EGR1 in the hippocampus of rat pups by RT-PCR. **E**–**G** The translational levels of CaMKIV, EGR3, and EGR1 by western blot. Experiments were performed in triplicates. Error bars represent the standard error (*P < 0.05).

CaMKIV has been found to localize within the neurons of CA1, CA3, and DG regions of the neonatal hippocampus, showing decreases in both translational and transcriptional levels in the condition of CH [9]. To address the potential regulatory role of CaMKIV on the EGR3, we examined the distribution of EGR3 protein in the neonatal hippocampus of CH rat pups. Immunostaining demonstrated that EGR3 colocalized with NeuN-positive neurons of hippocampal CA1, CA3, and DG (Fig. 4A). The expression abundance of EGR3 at translational and transcriptional levels in the hippocampus was significantly reduced by CH at PN1, PN7, and PN21, in parallel with those of CaMKIV (Fig. 4B, C, E, F). Contrarily, the expression of EGR1 was unaltered in the CH group compared to the control group (Fig. 4D, G). The data indicate that CaMKIV and EGR3 were downregulated in the hippocampus of CH rat pups.

### Knockdown of CaMKIV/EGR3 impaired the growth of dendritic spines

**Fig. 5:**
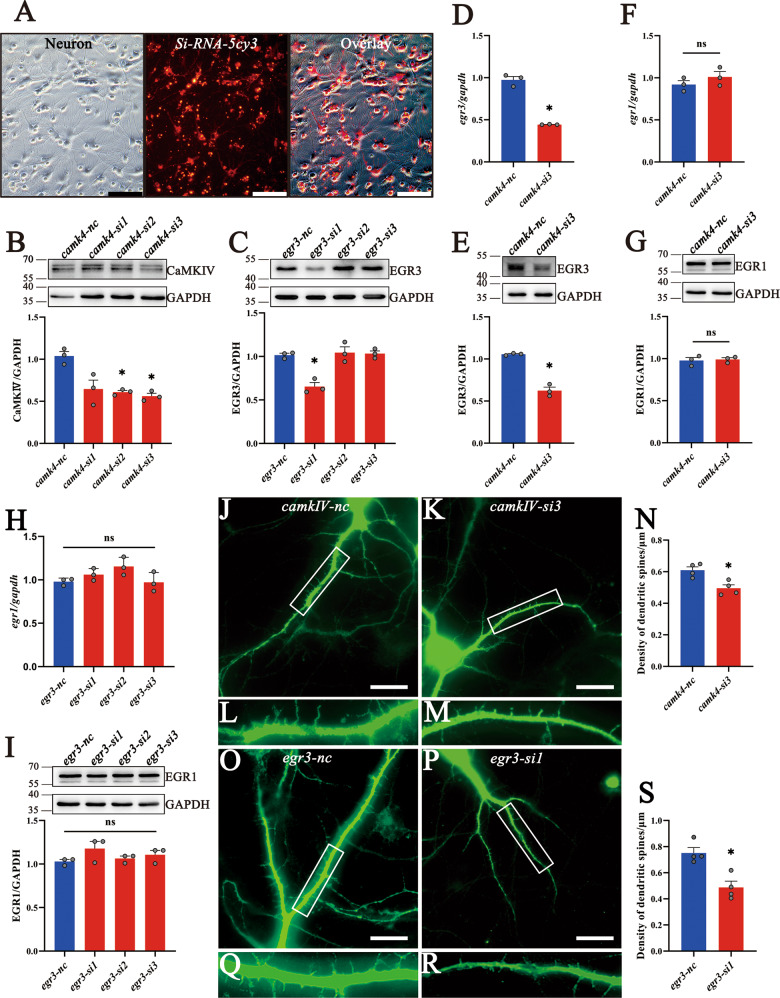
Knockdown of CaMKIV/EGR3 influences the growth of dendritic spines. **A** Transfection efficiency was evaluated by 200 nM Cy3-labeled siRNA oligonucleotide transfection in the primary hippocampal neurons for 24 h. Scale bar: 100 μm. **B**, **C** The interference efficiency of 200 nM siRNA oligonucleotides for CaMKIV (**B**) and EGR3 (**C**) was analyzed by western blot after cell transfection for 24 h. The *camk4-*Si3 and *egr3-*Si1 were used for the follow-up experiments. **D**–**G** Analysis of EGR3 (**D**, **E**), EGR1 (**F**, **G**) levels following CaMKIV knockdown of primary hippocampal neurons for 24 h by RT-PCR and western blot. **H**, **I** Analysis of EGR1 levels following EGR3 knockdown of primary hippocampal neurons for 24 h. Experiments were performed in triplicates. **J**–**M**, **O**–**R** The primary hippocampal neurons were knocked down with *camk4-*Si3 and *egr3-*Si1 and cultured for 5 days. The density of dendritic spines was detected by immunofluorescence staining with MAP2 antibody. The rectangle indicates the magnified region. **N**, **S** Statistical analysis of spine density in quadruplicates every 15 fields. Scale bar: 20 μm. Error bars represent the standard error (*P < 0.05).

The siRNA oligonucleotides were synthesized and applied to knockdown the expression of CaMKIV or EGR3 to further validate the regulatory role of CaMKIV on the EGR3 expression and their effects on the dendritic spine growth of hippocampal neurons. The transfection efficiency was evaluated by Cy3-labeled siRNA, which reached 87.3% in the primary hippocampal neurons after transfection with 200 nM siRNA oligonucleotides for 24 h (Fig. 5A). The siRNA3 for CaMKIV (*camkIV-*si3) and siRNA1 for EGR3 (*egr3-*si1) were chosen for their highest interference efficiency (Fig. 5B, C). Notably, *egr3*-si1 oligonucleotides displayed specificity without influencing EGR1 expression (Fig. 5H, I). Results revealed that the knockdown of CaMKIV in primary hippocampal neurons from E18 for 24 h decreased the expression of EGR3, rather than EGR1, at translational and transcriptional levels (Fig. 5D–G). Immunostaining of microtubule-associated protein 2 (MAP2) showed that interference of EGR3 expression with *egr3*-si1 in the primary hippocampal neurons markedly decreased the density of dendritic spines (to 64.9%), similar to those of CaMKIV knockdown (to 81.2%) (Fig. 5J–S). The data indicate that CaMKIV affects the dendritic spine growth of hippocampal neurons through EGR3 in CH rat pups.

### CaMKIV/EGR3 signal axis mediates deficient dendritic spine growth of hippocampal neurons through regulation of BDNF in the CH rat pups

**Fig. 6:**
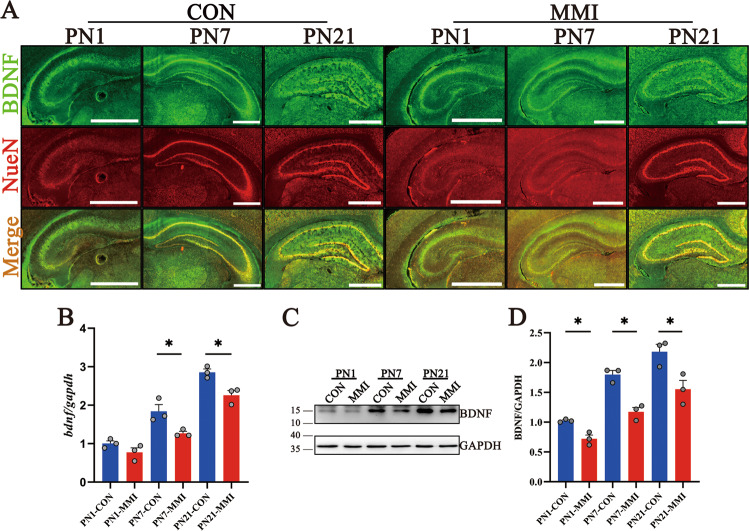
CH induced downregulation of BDNF expression in rat hippocampus. **A** The co-localization of BDNF with NeuN-positive neurons in the hippocampus by immunofluorescence staining. Scale bar: 1000 μm. **B**–**D** Transcriptional and translational levels of BDNF in the hippocampus of rat pups by RT-PCR and western blot. Experiments were performed in triplicates. Error bars represent the standard error (*P < 0.05).

**Fig. 7:**
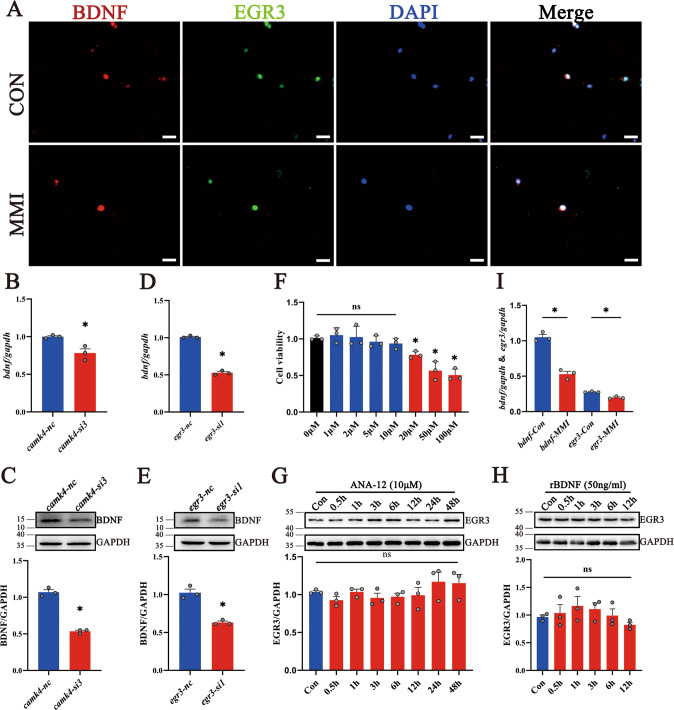
Knockdown of CaMKIV/EGR3 downregulated BDNF expression in primary hippocampal neurons. **A** Primary hippocampal neurons were cultured from E18 fetal rats with or without CH. The co-localization of BDNF and EGR3 was observed by immunofluorescence staining. Scale bar: 20 μm. **B**–**E** Analysis of BDNF levels following CaMKIV and EGR3 knockdown of primary hippocampal neurons for 24 h by RT-PCR (**B**, **D**) and western blot (**C**, **E**). **F** The cell viability of primary hippocampal neurons treated with different concentrations of ANA-12 for 24 h was detected by CCK8 kit. The cell viability decreased significantly when ANA-12 concentration was higher than 10 μM. **G** Expression of EGR3 in primary neurons treated with ANA-12 10 μM for the different times by western blot. **H** Expression of EGR3 in primary neurons treated with recombinant protein BDNF 50 ng/ml for the different times by western blot. **I** Transcriptional levels of EGR3 and BDNF in primary neurons cultured from hippocampus with or without CH by RT-PCR. Experiments were performed in triplicates. Error bars represent the standard error (*P < 0.05).

Now that EGR3 can promote BDNF expression through binding to the promoter of BDNF [11, 34–36], it is conceivable that CaMKIV/EGR3 mediates abnormal dendritic spine growth of hippocampal neurons through regulation of BDNF. To clarify the potential regulatory mechanism, we examined the expression of BDNF in the hippocampal neurons of CH rat pups. Results exhibited that BDNF was detected in the NeuN-positive hippocampal neurons at CA1, CA3, and DG regions (Fig. 6A), but its expression abundance at both transcriptional and translational levels were significantly reduced by CH, as shown by RT-PCR and Western blot analysis at PN1, PN7, and PN21 (Fig. 6B–D). Next, the primary hippocampal neurons from E18 rats of the CH and control group were cultured. Immunostaining showed that BDNF colocalized with EGR3 in the primary hippocampal neurons (Fig. 7A), and both had a decreased abundance in the CH group (Fig. 7I). Knockdown of CaMKIV or EGR3 expression for 24 h with *camkIV-*si3 or *egr3*-si1 significantly reduced the transcriptional and translational expression of BDNF in the primary hippocampal neurons from E18 (Fig. 7B–E). However, adding 50 ng/ml recombinant BDNF protein for 0–12 h or 10 μM TrkB receptor blocker ANA-12 for 0–48 h to the culture medium had no effects on the EGR3 expression of the primary hippocampal neurons (Fig. 7F–H). These data indicate that CaMKIV/EGR3 axis regulates BDNF expression in the hippocampal neurons of rat pups.

**Fig. 8:**
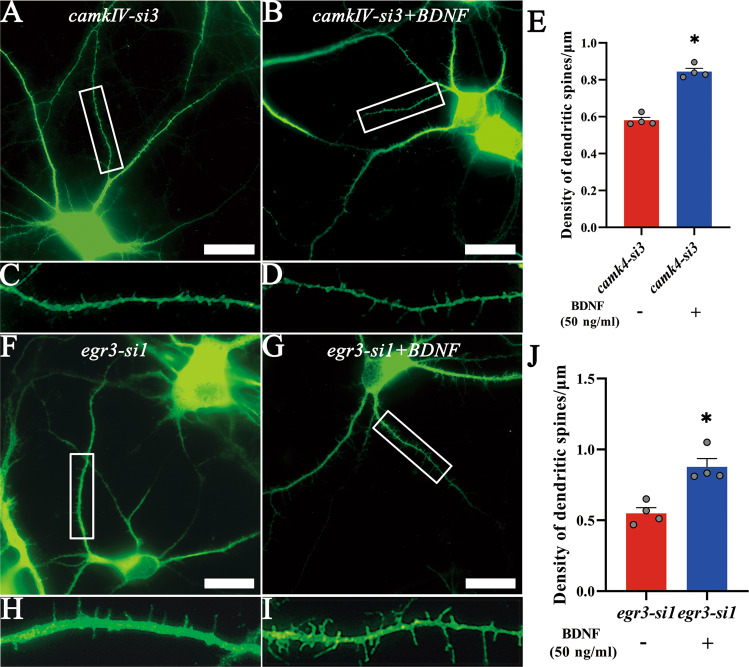
BDNF rescued the growth of dendritic spines following the knockdown of CaMKIV/EGR3. **A–D**, **F**–**I** The primary hippocampal neurons were transfected with *camk4-*Si3 and *egr3-*Si1 and cultured for 5 days, then supplemented with 50 ng/ml BDNF for 24 h, the density of dendritic spines was detected by immunofluorescence staining with MAP2 antibody. The rectangle indicates the magnified region. **E**, **J** Statistical analysis of spine density in quadruplicates every 15 fields. Scale bar: 20 μm. Error bars represent the standard error (*P < 0.05).

To explore whether BDNF can rescue the effects of CH-mediated deficient dendritic spine growth of hippocampal neurons regulated by aberrant CaMKIV and EGR3, the expression of CaMKIV or EGR3 in primary hippocampal neurons was knocked down with *camkIV-*si3 or *egr3*-si1 for 24 h, followed by addition of 50 ng/ml recombinant BDNF to the culture for another 24 h. Immunostaining of MAP2 demonstrated that BDNF successfully rescued the effects of CaMKIV and EGR3 dysregulation on the dendritic spine growth of the hippocampal neurons (Fig. 8A–J). The data indicate that CaMKIV/EGR3 signal axis mediates abnormal dendritic spine growth of hippocampal neurons through the regulation of BDNF in the CH rat pups.

## Discussion

CH is closely associated with the pathogenesis of thyroid dysplasia, TH anabolism disorder, TH resistance, and thyroid axis dysfunction [37–40], which influence intellectual and physical development [41]. Hippocampus is one of the most affected tissues by CH, leading to severely impaired cognitive function [9, 42, 43]. In this study, CH reduced the dendritic spine density of hippocampal neurons, whereas it did not affect the number of neurons in the DG region. It is known that dendritic spines are the main structure responsible for synaptic signal transmission [44–47], and their number and density are correlated with synaptic plasticity, learning, and memory function [34]. The dendritic spines are formed postnatally, showing a sharp increase in spine density in the hippocampal CA1 region of rats from PN1 to PN12 [48]. As such, CH-induced cognitive impairment may occur postnatally, and an immediate supplement of TH in neonates should be beneficial for alleviating late functional sequelae.

Propylthiouracil (PTU) and MMI are commonly used to establish the CH model of rat offspring [9, 49]. Continuous treatment of MMI on dams has successfully induced fetal hypothyroidism, confirmed by the TSH and T4 levels in serum [9, 50, 51]. We previously displayed that CH resulted in a loss of the body weight of rat pups [9], suggesting a role of CH on growth retardation other than intellectual disability [52, 53]. It was interesting to note that the brain volume of CH pups was significantly reduced in comparison with the control, whereas the morphology of the hippocampus and the number of neurons in hippocampal DG were unaltered. The possibility of CH affecting the proliferation and differentiation of the neural progenitor cells in the cortex cannot be excluded, as congenital hypothyroidism correlates with brain cortex immaturity [54].

As an important Ca^2+^ signaling pathway regulator, T3 potently induces CaMKIV expression during the early stages of brain development [7, 55]. Decreased T3 levels due to CH will lead to the inactivation of CaMKIV in the brain neurons, which causes the aberrancy of cortical connectivity and neocortical function [6, 56], as well as dendritic growth of hippocampal neurons [9]. EGR3 has been shown to be regulated by T3 in the glial cells of the peripheral nervous system (PNS) [57], but less information is available regarding T3-mediated EGR3 dysregulation in the CNS. Nevertheless, EGR3 has widely been implicated in multiple neuropathogenesis of CNS [58]. By transcriptome analysis, we herein revealed that EGR3 was under the control of CaMKIV to participate in CH-induced dendritic spine abnormality of hippocampal neurons, suggesting the universal regulatory mechanism of T3 on EGR3 in both CNS and PNS. CaMKIV activation is able to promote the phosphorylation of CREB [59, 60], which in turn promotes a set of downstream genes in multiple cell types, including EGR3 [61–63]. CaMKIV-mediated dendritic spine abnormality of hippocampal neurons in the context of CH is possibly involved in the indirect regulation of EGR3. However, the detailed regulatory mechanism deserves further study.

The neurotrophin BDNF plays an essential role in brain development and regular physiological activity [64]. Dysregulation of BDNF influences neurite development, synapse formation, and stabilization of CNS neurons, which are associated with multiple diseases of CNS, even postnatal death [65–67]. BDNF is highly expressed and released in the cerebral cortex, hypothalamus, striatum, cerebellum, and especially in the hippocampus of rodents [68–70]. T3 is one of the inducers of BDNF, and the absence of TH induces a developmental delay in primary hippocampal neurons through a decreased BDNF expression [71]. However, the underlying mechanism of T3 regulation on BDNF remains elusive. In the present study, CH-mediated failure of dendritic spine growth of hippocampal neurons was attributed to the dysregulation of the CaMKIV/EGR3 signal axis, which impacted the BDNF expression. Our results have unveiled the critical regulatory mechanism of CH-mediated cognitive impairment in the offspring, which might be helpful for the drug development of intellectual disability caused by CH.

In conclusion, CH of rat pups resulted in a decreased level of CaMKIV in the hippocampal neurons, which reduced the expression of EGR3, thereby inhibiting the production of BDNF and eventually led to the failure of dendritic spine growth of hippocampal neurons.

## Materials and methods

### Animals

Adult female Sprague–Dawley (SD) rats weighing 180–220 g were provided by the Experimental Animal Center of Nantong University. All animal experiments were approved by the Animal Ethics Committee of Nantong University, which adhered to the National Institute of Health guidelines on the protection and use of laboratory animals. Female SD pregnant rats and their pups were placed in standard cages and exposed to light and dark cycles for 12–12 h at 22 ± 2 °C, with free access to clean water and standard food.

### Establishment of CH model of rat pups

Pregnant rats were randomly divided into the MMI treatment group and the control group. SD rats were continuously given 0.02% MMI in drinking water from embryonic day 9 (E9) to postnatal day 21 (PN21) in the MMI treatment group. The control group was given normal clean drinking water. Serum TSH and T4 were measured on the E18 of pregnancy and PN1, PN7, and PN21 of offspring to verify the success of the CH model.

### HE staining

The brain tissues of PN1, PN7, and PN21 offspring rats with or without CH were dissected after the successful establishment of the CH model and then quickly put into 4% paraformaldehyde and fixed at 4 °C for 24 h. After fixation, samples were gradually dehydrated with alcohol, treated with xylene, embedded with paraffin, and finally cut into 5-μm thick slices for standby. The tissue of a neutral gum patch was used to observe the morphology of the brain under a microscope using hematoxylin and eosin staining.

### Nissl’s staining

The brain tissue slices were immersed in toluidine blue dye for 5 min, rinsed with water, and separated color with 1% glacial acetic acid. Finally, sections were sealed with neutral glue, and hippocampal neurons were observed under the microscope.

### Golgi–Cox staining

Golgi staining was performed according to the instructions of the Hito Golgi staining kit (Hitobiotech, Beijing, China). The hippocampus of PN21 rat pups was immersed in solution A and solution B at room temperature for 2 weeks and then immersed in solution C at 4 °C for 48 h. The hippocampus was sectioned into 100-μm and pasted on 2% gelatin slide. The slices were dried at room temperature for 2 days, dyed with a mixture of solution D, solution E, and distilled water (1:1:3) for 10 min, and then dehydrated with gradient ethanol. Finally, neutral gum was used to seal the sections after treatment with xylene, and the density of hippocampal dendritic spines was observed under a microscope.

### Primary culture of hippocampal neurons, siRNA transfection, and drug treatment

Cultures of hippocampal neurons were performed as previously described [72]. In brief, the E18 SD rats were placed in a sealed anesthesia container with cotton balls thoroughly infiltrated with ether. The pregnant rats were sterilized by soaking in 75% ethanol after the corneal reflex and pain reflex disappeared, and the newborn rats were removed by laparotomy. Fetal rats were decapitated, and the hippocampus was isolated and placed in ice-cold Hank’s Balanced salt solution (HBSS), followed by digestion with 3 ml of 0.125% trypsin at 37 °C for 15 min. A high glucose medium containing fetal bovine serum was added to terminate digestion. The cells were centrifuged at 1000 rpm for 4 min, and the supernatant was removed. Then 2 ml medium at 37 °C was added to resuspend the cells. For primary culture, hippocampal neurons (1 × 10^4^ cells/cm^2^) were planted in 6- or 24-well plates embedded with poly-d-lysine, with or without small round glass slides. The original culture medium was discarded and replaced with the neural basal neuron culture medium containing B27 following 4–6 h of culture. The cells were cultured at 37 °C, 5% CO_2_, and 95% air atmosphere. The fresh medium was replaced every 2–3 days.

The siRNA transfection of the primary neurons was performed according to the manufacturer’s protocol (RiboBio, Guangzhou, China). 20 μl of 20 μM siRNA was added to 120 μl 1× riboFECT™ CP buffer, and then 12 μl riboFECT™ CP reagent was added and incubated at room temperature for 15 min; the transfection complex was transferred to a 2 ml neural basal medium containing cultured primary neurons. The final concentration of siRNA was 200 nM. Neurons continued to be cultured at 37 °C in 5% CO_2_ and 95% air for 24 h. The protein was extracted to determine the corresponding siRNA transfection efficiency.

For drug treatment of primary cultured neurons, the BDNF recombinant protein (Peprotech, New Jersey, USA) or TrkB receptor blocker ANA-12 (AbMole, Chicago, USA) was added into the culture medium at a final concentration of 50 ng/ml or 10 μM for 24 h, respectively.

### Western blot

Proteins were extracted from the cultured neuron or hippocampus with RIPA lysis buffer (MCE, New Jersey, USA) containing PMSF. The BCA method evaluated the protein sample concentration for the same loads. The protein was denatured by heating at 95 °C for 5 min, separated by electrophoresis on 10% SDS-PAGE, and transferred to the PVDF membrane. PVDF membrane was put into 1:1000 primary antibody solution and incubated at 4 °C overnight. The next day, PVDF membrane and 1:1000 diluted secondary antibody buffer were incubated at room temperature for 2 h (goat-anti-rabbit or donkey-anti-mouse HRP, Proteintech). Finally, the ECL kit was used to detect the Western blot band when the PVDF membrane was cleaned. Antibodies used for western blot were: anti-CaMKIV antibody (1:1000; Santa Cruz); anti-EGR3 antibody (1:1000; Santa Cruz); anti-BDNF antibody (1:1000; Santa Cruz); anti-GAPDH antibody (1:1000; CST).

### Transcriptome sequencing and bioinformatics analysis

According to instructions, total RNA was extracted from PN1, PN7, and PN21 pups using the mirVana miRNA isolation Kit (Ambion, Austin, TX). RNA purification beads (Illumina, San Diego, CA) were used to screen mRNA from the total RNA. IlluminaTruSeqRNA sample PrepKitv2 was used to construct the library, which was sequenced by IlluminahisEQ-2000 for 50 cycles and stored by Illumina quality filter for sequence analysis. The differentially expressed mRNAs were specified as standard for more or less than a twofold change compared with controls. Blastx results were utilized to annotate the gene function, which referred to NCBI or AGRIS database (https://agris-knowledgebase.org/), the *E*-value threshold of this approach was set to 10^−5^. For all heatmaps, genes were clustered by Jensen–Shannon divergence. Ingenuity pathway analysis (IPA) software was used to reconstruct a gene network based on differentially expressed genes to study their regulatory pathways and cellular functions [73].

### Immunofluorescence staining

The brain tissues of PN1, PN7, and PN21 pups were quickly dissected and fixed in 4% paraformaldehyde at 4 °C for 24 h. Then the brain tissues were dehydrated in 10%, 20%, and 30% sucrose solutions before being cut into 12-μm slices by a frozen slicer. After drying in an oven at 37 °C, the brain slices were stored at −20 °C. Primary neurons were grown on small round glass slides for cell immunofluorescence staining. Sections were rewarmed and washed in PBS for 5 min three times and sealed with PBS containing 0.1% TritonX-100, 5% fetal goat serum, and 5% horse serum at 37 °C for 1 h. The primary antibody diluted in PBS was added to slides for incubation overnight at 4 °C. The secondary antibodies (donkey-anti-mouse Alexa488 and goat-anti-rabbit Cy3) were added for incubation at 4 °C for 16 h. Finally, the sections were sealed with an anti-fluorescence quenching solution containing DAPI and observed under Zeiss Axio Image M2 microscope. Antibodies used were: anti-NeuN antibody (1:500; Sigma); anti-CaMKIV antibody (1:200; Santa Cruz); anti-EGR3 antibody (1:200; Santa Cruz); anti-BDNF antibody (1:200, Santa Cruz) anti-MAP2 (1:500; Abcam); donkey-anti-mouse Alexa488 and goat-anti-rabbit Cy3.

### Q-PCR

Total RNA was extracted from the hippocampus or cultured neurons with TRIzol (Gibco). The cDNA was diluted 1:4 for Q-PCR detection. Sequence-specific primers were designed and synthesized by Generay (Shanghai, China). The details are as follows: for CaMKIV, forward primer 5’-TGG AGG CAG TTG CTT ACC TG-3’ and reverse primer 5’-CCT CGG AGA ATC TCA GGT GC-3’; for EGR3, forward primer 5’-CTC AGA TGG CTA CAG AGA ATG TG-3’ and reverse primer 5’-ACC AGT TGG AAG GAG AGT CG-3’; for BDNF, forward primer 5’-GTC CCG GTA TCA AAA GGC CA-3’ and reverse primer 5’-ATC CTT ATG AAC CGC CAG CC-3’; for GAPDH, forward primer 5’-ACA GCA ACA GGG TGG TGG AC-3’ and reverse primer 5’-TTT GAG GGT GCA GCG AAC TT-3’. The reaction was subjected to an initial denaturation cycle at 94 °C for 5 min and then denaturation at 94 °C 30 s, 60 °C 30 s, and 72 °C 30 s for 45 cycles, respectively. The fluorescence signal strength was recorded at each annealing step. At the end of each PCR run, the system will automatically analyze the data and get the amplification map. The expression levels of these genes were normalized to an endogenous *gapdh* cDNA.

### Statistical analysis

All statistical tests were performed in GraphPad Prism 8.0 (California, USA). Levene’s test was used to detect the normal distribution of all data, Student’s *T* test was used for individual comparison of two data, and one-way analysis of variance (ANOVA) followed by Bonferroni’s post hoc test was utilized for multiple comparisons. Bilateral *P* values < 0.05 were considered to be statistically significant.

## Supplementary information

figS1

tableS1

supplementary legends

## Data Availability

The datasets used and/or analyzed during this study are available from the corresponding author on reasonable request.
