# Supplementary figures and images for: CaMKIV mediates spine growth deficiency of hippocampal neurons by regulation of EGR3/BDNF signal axis in congenital hypothyroidism
Source: Cell Death Discov. 2022 Dec 6;8:482. doi: 10.1038/s41420-022-01270-4 (PMC9723595; doi:10.1038/s41420-022-01270-4)

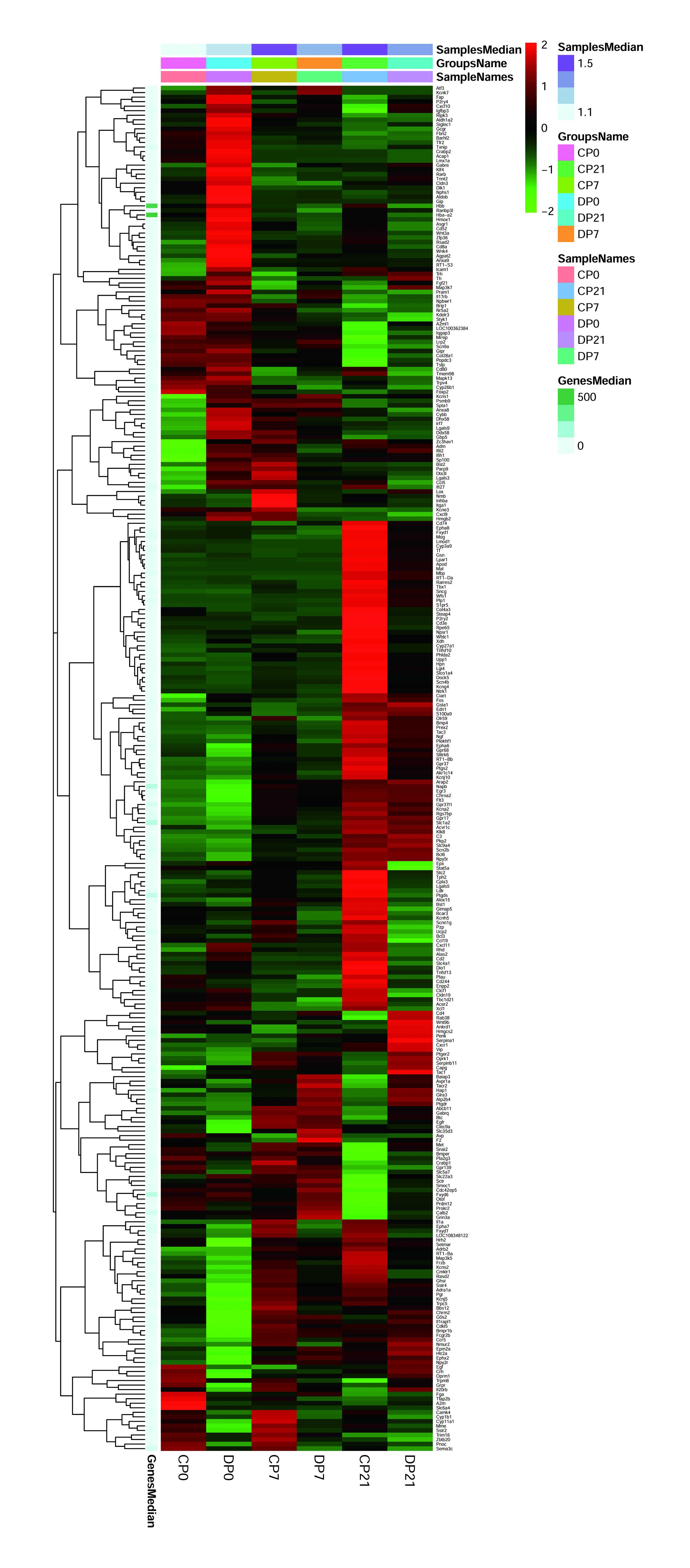

Supplement: Supplementary file 1 — figS1 [file 41420_2022_1270_MOESM1_ESM.tif]
