# Supplementary material for: CaMKIV mediates spine growth deficiency of hippocampal neurons by regulation of EGR3/BDNF signal axis in congenital hypothyroidism
Source: Cell Death Discov. 2022 Dec 6;8:482. doi: 10.1038/s41420-022-01270-4 (PMC9723595; doi:10.1038/s41420-022-01270-4)
Supplement: Supplementary file 3 — supplementary legends [file 41420_2022_1270_MOESM3_ESM.docx]

**TableS1**. Differentially expressed genes analyzed by transcriptome sequencing of hippocampal tissues following rat CH at PN1, PN7 and PN21.

**FigureS1**. Heatmap and cluster dendrogram of DEGs in the hippocampus of P1, P7 and P21 rat pups following CH.
